# Supplementary material for: MetaRibo-Seq measures translation in microbiomes
Source: Nat Commun. 2020 Jun 29;11:3268. doi: 10.1038/s41467-020-17081-z (PMC7324362; doi:10.1038/s41467-020-17081-z)
Supplement: Supplementary file 10 — Supplementary Data 7 [file 41467_2020_17081_MOESM10_ESM.zip › File2/Confidence_High_Taxonomy/386898_out.krona.html]

Javascript must be enabled to view this page.

members
magnitude
magnitudeUnassigned
count
unassigned
taxon
rank

386898\_out

68

2
superkingdom
68

68
phylum
1239

186801
class
68

186802
order
68

35
family
541000

1263
genus
35

2293214

SRS015217\_contig\_number\_18255SRS015782\_contig\_number\_42974SRS019068\_contig\_number\_47074SRS042628\_contig\_number\_32707SRS048870\_contig\_number\_12725SRS1041091\_contig\_number\_20336
6
species

2293187
species
1

SRS014979\_contig\_number\_13558


SRS012273\_contig\_number\_45647SRS013638\_contig\_number\_8373SRS014235\_contig\_number\_28432SRS015578\_contig\_number\_contig-100\_1826.184846SRS015663\_contig\_number\_4122SRS015854\_contig\_number\_2461SRS015890\_contig\_number\_25932SRS018984\_contig\_number\_29243SRS019601\_contig\_number\_29579SRS019685\_contig\_number\_42801SRS020233\_contig\_number\_5745SRS024492\_contig\_number\_16330SRS048060\_contig\_number\_6536SRS048164\_contig\_number\_11170SRS050752\_contig\_number\_29548SRS051031\_contig\_number\_24105SRS052697\_contig\_number\_34635SRS062701\_contig\_number\_6424SRS098571\_contig\_number\_29848SRS104197\_contig\_number\_327SRS142542\_contig\_number\_contig-100\_925.162795SRS143991\_contig\_number\_1597SRS146764\_contig\_number\_25261SRS147022\_contig\_number\_10207SRS147766\_contig\_number\_8561SRS148159\_contig\_number\_16755
26
species
46228

species
2

SRS148511\_contig\_number\_21624SRS148721\_contig\_number\_32534
2293238

31
family
186803

1898203
species
1

SRS018313\_contig\_number\_9866

genus
1
189330

species
1

SRS049712\_contig\_number\_8992
1263074

genus

SRS148196\_contig\_number\_30522
1
33042

genus
27
572511

33039

SRS015578\_contig\_number\_30482SRS016095\_contig\_number\_27029SRS050422\_contig\_number\_31983SRS1041133\_contig\_number\_13871SRS104636\_contig\_number\_23576SRS144506\_contig\_number\_45606SRS144537\_contig\_number\_40860SRS147919\_contig\_number\_7979SRS148253\_contig\_number\_493
9
species

33038

SRS012273\_contig\_number\_25116SRS012902\_contig\_number\_contig-100\_920.920SRS014948\_contig\_number\_1523SRS015578\_contig\_number\_22351SRS017521\_contig\_number\_33357SRS042284\_contig\_number\_4896SRS054590\_contig\_number\_contig-100\_1231.1231SRS057717\_contig\_number\_2161SRS063518\_contig\_number\_contig-100\_997.204264SRS075398\_contig\_number\_contig-100\_796.29464SRS077502\_contig\_number\_17360SRS1041095\_contig\_number\_contig-100\_1495.35592SRS1055043\_contig\_number\_23406SRS1055067\_contig\_number\_contig-100\_2214.75767SRS148511\_contig\_number\_21622SRS149879\_contig\_number\_6333SRS893253\_contig\_number\_contig-100\_788.789SRS893259\_contig\_number\_contig-100\_1234.46779
18
species

1
genus
830

45851
species
1

SRS015431\_contig\_number\_48587

31979
2
family

1485
genus
2

1720194

SRS014287\_contig\_number\_16062
1
species


SRS098644\_contig\_number\_25572
1
species
1262777
